# Experimental Study on Vibration and Noise Characteristics of Steel-Concrete Railway Bridge

**DOI:** 10.3390/s21237964

**Published:** 2021-11-29

**Authors:** Lucjan Janas

**Affiliations:** Faculty of Civil and Environmental Engineering and Architecture, Rzeszów University of Technology, al. Powstańców Warszawy 12, 35-959 Rzeszów, Poland; ljanas@prz.edu.pl

**Keywords:** vibration assessment, noise assessment, noise sources, steel-concrete bridge

## Abstract

The paper presents the results of vibroacoustic tests of a plate girder railway bridge consisting of two parallel dilated structures and a common ballast trough. The requirements currently set for railway bridges relate to, among others, vibrations considered as one of the criteria for traffic safety and to noise emissions that may pose a threat to the environment. In this article, the results of tests conducted on vibrations of elements of the analyzed structure are presented, and the level of these vibrations in terms of meeting the requirements of the European standards is assessed. Vibrating criteria of structure performance were checked, and safety was assessed. The results of noise measurements in the vicinity of the analyzed bridge are also presented, and the environmental impact of this structure is determined. The test results show that the bridge meets the requirements for vibration acceleration and noise. An increased acoustic emission in the analyzed case does not pose a significant threat, but if this type of structure was on high supports in an urbanized area, it would be a nuisance to the environment.

## 1. Introduction

Experimental research for the assessment of railway bridges is important in the context of identifying their serviceability condition. Dynamic tests of bridges are used to assess behavior under train loadings, validate design conceptions, evaluate condition state, carry out damage detection based on vibration analysis, and perform environmental impact assessment.

The Eurocode [1] includes serviceability limit states and other specific limit states for bridges. In section A.2.4.4.2, which is devoted to “Criteria for traffic safety”, it is stated that in order to ensure safety, the acceleration of span vibrations should be checked. In this standard, it was also noted that the recommended maximum values of span acceleration, determined along each track, should not exceed 3.5 m/s^2^ in the case of tracks laid on ballast. The issue of bridge vibrations has been raised in many research papers, including [2,3,4,5,6].

In addition to the vibration issue, in the European standards also the issue of noise generated by bridges has been highlighted. In the Eurocode [7], in the chapter on serviceability limit states, in the general provisions section, it is stated that the natural frequency of vibrations of the structure should be limited, among others, to reduce excessive noise emissions. In the section regarding the performance criteria for railway bridges, it is stated that all requirements for the limitation of noise emission may be given in the project specification. It should be noted that the main sources of railway noise are wheel and rail friction as well as engine and equipment (e.g., brakes) noise. At higher speeds, aerodynamic noise also occurs. Trains crossing bridges, viaducts, or flyovers may have a much greater impact on the environment than trains using a track without engineering structures. This impact may be manifested by increased noise emissions. General recommendations for designing silent bridges are given in [8]. In this study, among other things, there is a general recommendation that ballasted track should be used on bridges where there are noise problems. If necessary, an additional mat may be inserted under the ballast to provide an extra reduction of noise. The increase of noise emissions depends primarily on the bridge type, for example for steel bridges with open deck, and concrete bridges with direct rail fastenings noise reaches even up to 15 dB [9,10,11]. The study [12] stated that when a train passes over a bridge, vibrations are generated owing to irregularities in the wheels and the track. These vibrations cause the wheels and track to radiate noise and transfer energy directly to each component of the bridge, causing the beams and other components to vibrate, thus forming secondary noise radiation. The magnitude of such bridge-borne noise can typically be 10 dB or more for common railway networks. The greatest threats to the environment are steel structures without crushed stone ballast [13,14] as well as those without vibroinsulation. However, structures with ballast may also affect the deterioration of the acoustic climate in the vicinity of a railway line. Concrete, prestressed concrete, and composite bridges generally cause far fewer acoustic problems than steel bridges, although such problems cannot be excluded [15]. The issue of railway bridge noise has also been raised, among others, in [16,17]. Detailed identification of the sound sources is required to reduce noise. An example for a steel bridge with an orthotropic plate is presented in [18]. The problem of noise reduction around railway lines and bridges was raised, among others in [19,20,21].

In this paper, the results of the vibration and noise analysis of a new railway bridge of a relatively unusual, unique structure are presented. Vibracoustic properties of this type of construction have not yet been presented in the literature. The purpose of the tests was to check whether this type of structure meets the vibration and noise emission requirements and to identify possible noise sources. In order to check whether the bridge has a negative impact on the environment, the simultaneous measurement of accelerometers and microphones was used, and the coherence function was determined.

## 2. Structure of the Analyzed Bridge and Measurement System

**Figure 1:**
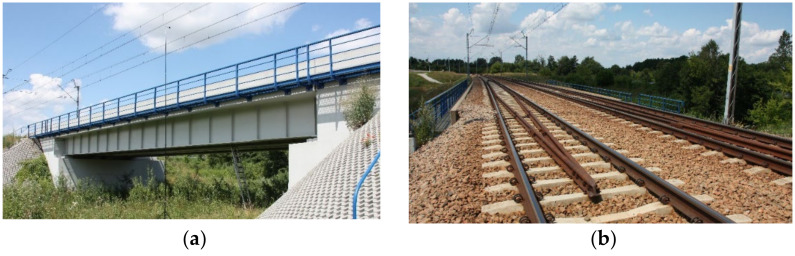
Side (**a**) and top (**b**) view of the tested bridge.

The structure subjected to a detailed vibroacoustic analysis was a plate girder railway bridge, located along the main line with a maximum train speed of 160 km/h. The bridge superstructure was designed as a single-span, simply supported, composite, steel-concrete structure. Side and top views of the bridge are shown in Figure 1. In the cross-section of the structure, a monolithic reinforced concrete slab was used with the thickness varying from 0.23 m above the supports to 0.25 m in the middle of the span. The slab was connected with steel girders with the height ranging from 1.82 m in the middle of the span to up to 1.56 m above the supports.

The girders were made of S355J2 steel, whereas the deck was made of C35/45 concrete. The bridge consists of two parallel structures connected by a dilatation joint, with one ballast trough. The longitudinal dilatation gap was secured with flexible sealing tape and galvanized steel sheet with the thickness of 4 mm. The ballast trough was lined with a waterproof membrane with a 12 mm thick protective layer, resistant to crushed stone ballast. There was no vibroisolation under the ballast. The railway track on the bridge consists of UIC60 rails with 49E1 check rails, PS94 concrete sleepers laid at a spacing of 0.55 m, and the ballast in the form of 0.35 m thick crushed stone placed under the sleepers. The rails were attached to the sleepers with a SB-type elastic fastening. Service decks were built on both sides of the bridge, with the usable width of 0.75 m. The theoretical span was 25.55 m and total width amounted 10.62 m. The object technical condition during the test was good.

Accelerometers were used to measure the maximum vibrations. In order to determine if additional acoustic effects appear along the section on which the analyzed bridge is located, acoustic pressure measurements were carried out.

The following measurement system was used to evaluate the bridge:

• 6-channel sound and vibration data acquisition module,
• uniaxial (1 axis) piezoelectric Brüel&Kjær accelerometers with sensitivity of 50 mV/ms^2^, measuring range of 0.2 to 6000 Hz and ±140 m/s^2^,
• multi-field Brüel&Kjær microphones with a 20 dB(A) noise floor, a maximum SPL of 130 dB, and optimized frequency response 12–20,000 Hz,
• accelerometers calibrator, reference sound source and weather station.

**Figure 2:**
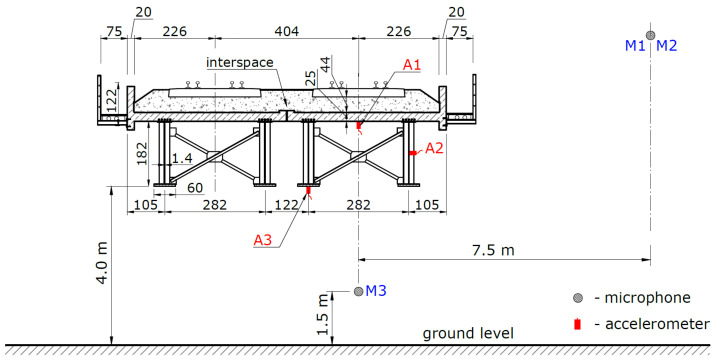
Cross-section of the tested bridge and measuring points.

The location of the measurement points is shown in Figure 2. Accelerometers were mounted on the deck slab (m.p. A1), girder web (m.p. A2), and bottom flange (m.p. A3).

A set of microphones was used at three following measuring points:

• at the distance of 7.5 m from the track axis and 1.5 m above the rail level but at a distance of 50 m beyond the bridge (m.p. M1),
• next to the bridge, at the distance of 7.5 m from the track axis and 1.5 m above the rail level (m.p. M2),
• under the bridge, 1.5 m above the ground level (m.p. M3).

**Figure 3:**
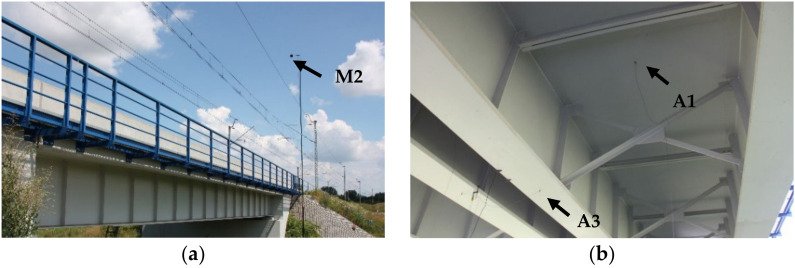
Measurements points: (**a**) microphone at point M2, (**b**) accelerometers mounted on the deck (m.p. A1) and the bottom flange of the girder (m.p. A3).

Selected measurement points are shown in Figure 3.

The microphones were placed in such a way as to check the noise differences in the vicinity of the bridge in relation to the noise next to the track outside the bridge. The identification of sound sources was limited to representative bridge elements with large surfaces. Only on such representative surfaces were accelerometers installed. In order to check whether the bridge deck plate causes sound emission, one of the microphones was placed under the bridge.

## 3. Vibration Research

**Figure 4:**
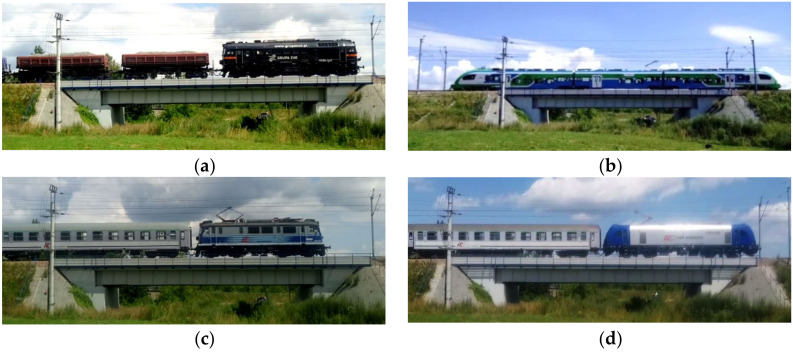
Images of chosen rail vehicles crossing the analyzed bridge: (**a**) cargo train with M62 locomotive, (**b**) railbus—PESA EN62A, (**c**) passenger train with EP-07 locomotive, (**d**) passenger train with NEWAG locomotive.

The experimental research allows the actual dynamic parameters of the structure to be determined. In total, ten train passages were analyzed. Among them, there were passenger and cargo trains (Figure 4). Natural vibration frequencies, acceleration amplitudes, and a logarithmic decrement of damping were determined. Train speeds were determined on the basis of video recordings.

**Figure 5:**
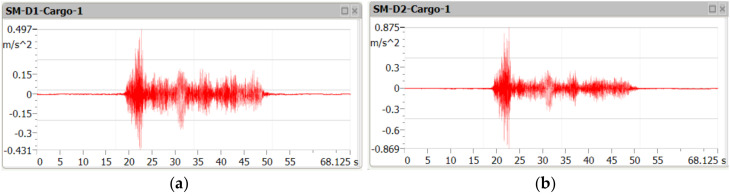
Amplitude of vertical vibration acceleration during the passage of the cargo train at the speed of 40 km/h, in the deck slab (**a**) and the bottom flange of the girder (**b**).

**Figure 6:**
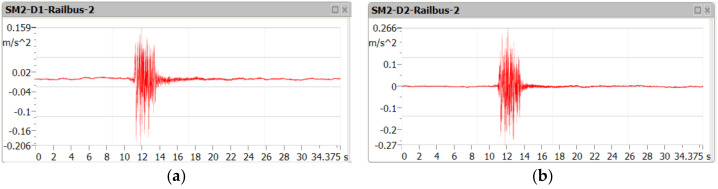
Amplitude of vertical vibration acceleration during the passage of the railbus at the speed of 115 km/h, in the deck slab (**a**) and the bottom flange of the girder (**b**).

**Figure 7:**
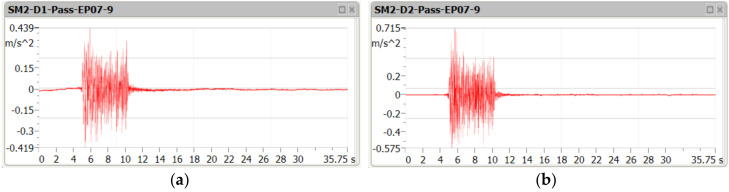
Amplitude of vertical vibration acceleration during the passage of the passenger train at the speed of 110 km/h, in the deck slab (**a**) and the bottom flange of the girder (**b**).

Figure 5, Figure 6 and Figure 7 contain examples of vertical vibration acceleration characteristics as a function of time recorded in the deck slab (m.p. A1) and the bottom flange of the girder (m.p. A3).

In Table 1, vibration accelerations recorded during the passages of ten trains are listed.

The maximum vertical acceleration value measured during the experimental tests was 0.87 m/s^2^. Such accelerations were recorded during the passage of a cargo train. The passage of railbuses causes vibrations not exceeding 0.27 m/s^2^. Passenger trains cause vibrations not greater than 0.71 m/s^2^. All these accelerations are significantly lower than the values allowed by the standard [1], i.e., 3.5 m/s^2^. It is worth noting that during the passenger train passage the vibration acceleration was lower, even though the speed was higher. It should be noted that when a train is passing one structure, vibration is damped by the adjacent structure via crushed stone.

## 4. Noise Research

The main sources of railway noise may be divided into four groups:

• noise arising from rolling stock; internal combustion and electric engines powering the locomotives, train equipment (compressors, brakes, pantographs etc.),
• noise arising from vibrations as well as wheel and rail friction,
• aerodynamic noise,
• noise arising from engineering structures.

In this article, the impact of the bridge structure on noise in the vicinity of the railway line was tested. The acoustic phenomenon was registered during the passage of the trains listed in Table 1 at the points shown in Figure 2. The microphones were placed in such a way as to check the noise differences in the vicinity of the bridge in relation to the noise next to the track outside the bridge The results of noise measurements are presented in Table 2. The reported noise values (L_Aeq_) were determined when the train was approaching the bridge (M1) and while it was crossing the bridge (M2, M3).

For the tested bridge, an increase of noise next to the bridge of about 1.1–5.8 dB was noticed. When passenger trains pass, the noise under the bridge is up to 4.6 dB higher than the noise next to the railway line outside the bridge. The reasons for the noise increase under the bridge may be vibrations from the vehicle wheels transmitted through the rails and ballast to the bridge structure. When passenger trains pass, the noise under the bridge is clearly lower than the noise next to the railway line and next to the bridge. The bridge has better acoustic properties than steel bridges, where the noise next to the bridge and under the bridge may increase by more than 15 dB [13]. Compared to the plate girder bridge with steel orthotropic platform and a track laid on ballast, next to the tested bridge the noise is higher, and under the bridge it is lower. More information can be found in [18].

**Figure 8:**
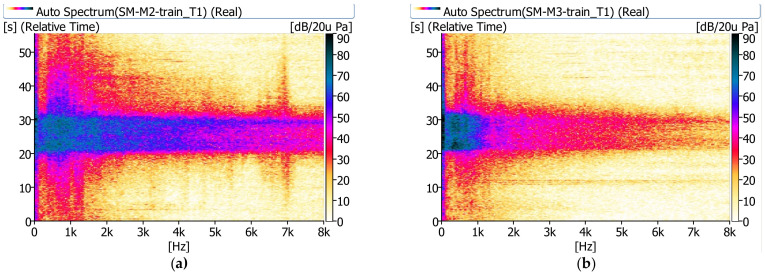
Spectrograms recorded during the passage of a cargo train (T1): (**a**) next to the bridge (m.p. M2), (**b**) under the bridge (m.p. M3).

**Figure 9:**
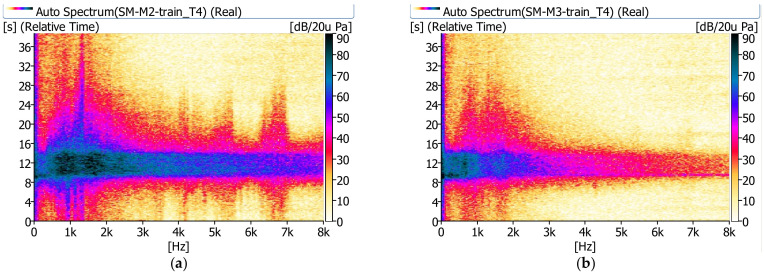
Spectrograms recorded during the passage of a passenger train (T4): (**a**) next to the bridge (m.p. M2), (**b**) under the bridge (m.p. M3).

**Figure 10:**
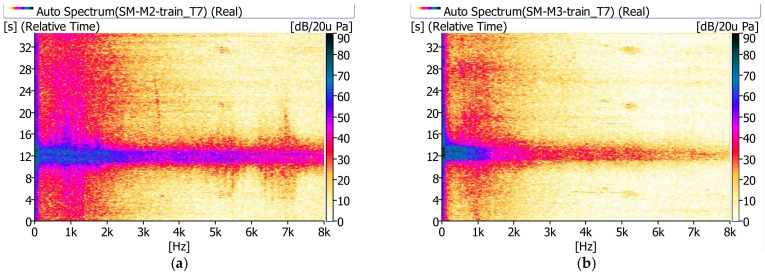
Spectrograms recorded during the passage of a railbus (T7): (**a**) next to the bridge (m.p. M2), (**b**) under the bridge (m.p. M3).

In Figure 8, Figure 9 and Figure 10, examples of measurement results in the form of spectrograms are shown.

The sound pressure level next to the bridge (m.p. M2) shows significant values in the range up to 8 kHz. A relatively high sound pressure level is observed longer besides the bridge than under the bridge. The noise under the bridge disappears almost immediately after the train passes. It is clearly visible that under the bridge (m.p. M3) the sound pressure level has significant values in the range up to approximately 1.0 kHz.

**Figure 11:**
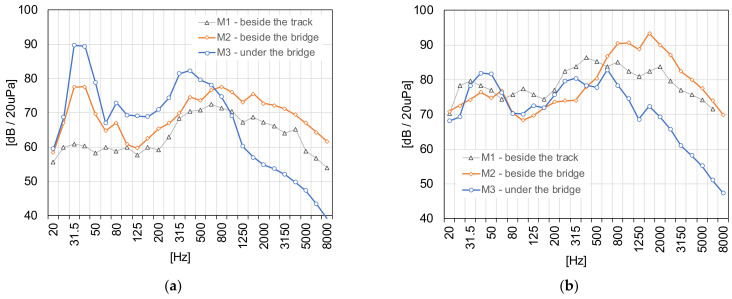
Levels of the acoustic pressure versus frequency bands during the passage: (**a**) cargo train (T1) at the speed of 40 km/h, (**b**) passenger train (T4) at the speed of 110 km/h.

Figure 11 shows examples of measurement results in the form of third-octave sound spectra.

In the case of the analyzed bridge, during the passage of a cargo train, the acoustic pressure level next to the bridge (m.p. M2) is larger in all frequency ranges than the level at the reference point, beside the track (m.p. M1). The acoustic pressure level under the bridge (m.p. M3) is larger in the range of 25–800 Hz than the level beside the bridge (m.p. M2) and lower than the acoustic pressure beside the track in high frequency (over 1000 Hz).

During the passage of a passenger train, the acoustic pressure level next to the bridge (m.p. M2) is greater in the range over 600 Hz than the level at the reference point, beside the track (m.p. M1). The acoustic pressure level under the bridge (m.p. M3) is lower above 400 Hz than the level beside the bridge (m.p. M2) and the lower than acoustic pressure beside the track in almost a whole frequency range. In order to explain the causes of such phenomena, vibroacoustic tests were carried out.

## 5. Study of Acoustic and Vibration Phenomena

The acoustic and vibration tests were carried out in order to determine the effect of bridge elements on sound emissions to the environment. If sounds are emitted in such structures, they arise mainly as a result of vibrations of large-surface elements [10,16]. Therefore, the identification of sound sources was limited to representative elements with large surfaces. The following elements were subjected to tests (Figure 2): the deck in vertical direction (m.p. A1) and the girder web in horizontal direction (m.p. A2).

Along with the vibration measurements of structure elements, the level of acoustic pressure next to the bridge (m.p. M2) and under the bridge (m.p. M3) was measured.

A coherence function was applied to identify the main sources of noise in the bridge structure. Subjecting the measured signals to the Fourier transform allowed an analysis of the signals in the frequency domain. The square of the standardized function of mutual correlation corresponded to the value of the standardised coherence function. A comparison of the coherence function values for various elements of the structure and directions allowed the main sources of acoustic energy radiation to be determined. The value of the coherence function can be lowered as a consequence of the interfering noise. In the current study, the coherence function was determined using the formula (1):

(1) $$\gamma_{xy}^{2}\left(f\right)=\frac{{\left|{\bar{G}}_{xy}\left(f\right)\right|}^{2}}{{\bar{G}}_{xx}\left(f\right)\;\cdot \;{\bar{G}}_{yy}\left(f\right)}$$

where *Gxy(f)* is the cross-spectral density between signals *x* (noise) and *y* (vibrations), while *Gxx(f)* and *Gyy(f)* denote the auto spectral density of *x* and *y*, respectively.

This method (i.e., determining the coherence functions of the ‘material’ vibration and acoustic vibration type) can be utilized for studying the effect of individual vibration sources on the acoustic pressure at a specific point of an acoustic field. The above-mentioned functions revealed that relationships existed between the vibration phenomena of structural elements and the acoustic phenomena.

**Figure 12:**
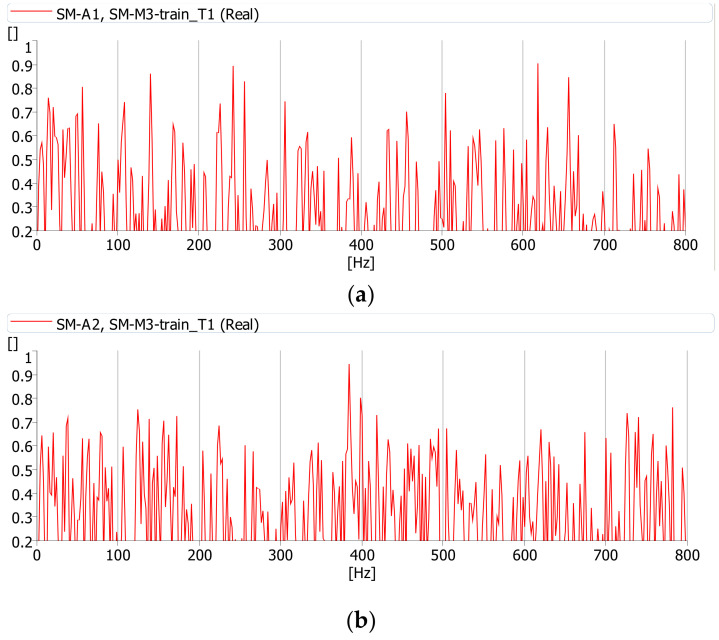
Coherence function between acoustic pressure at point M3 and: (**a**) vibrations at point A1 (deck slab), (**b**) vibrations at point A2 (girder web) during the passage of a cargo train (T1).

**Figure 13:**
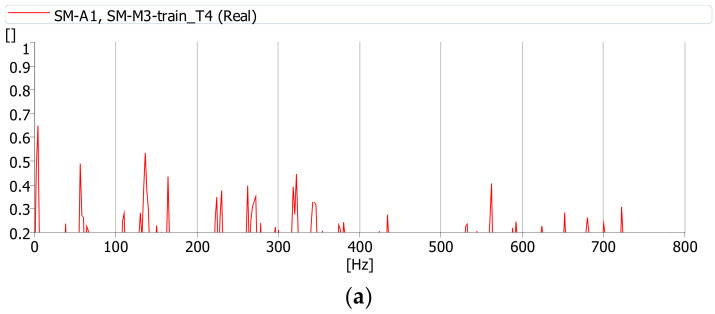

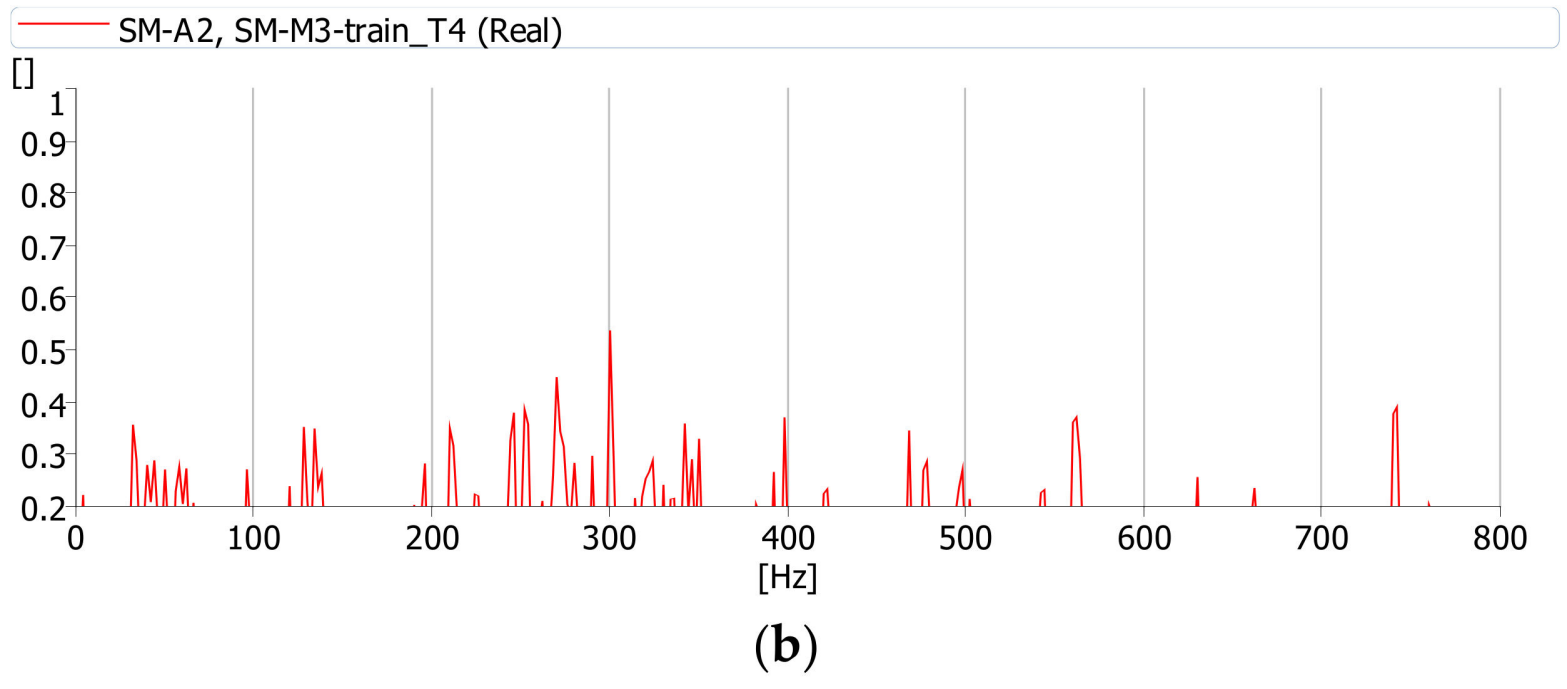
Coherence function between acoustic pressure at point M3 and: (**a**) vibrations at point A1 (deck slab), (**b**) vibrations at point A2 (girder web) during the passage of a passenger train (T4).

The coherence function was calculated during the journey of a cargo and passenger train. All functions were determined with the same parameters (averaging time, frequency range, etc.). This function has the highest values between the deck (m.p. A1) and girder web (m.p. A2) vibrations and noise (m.p. M3) while a cargo train passes (Figure 12). Therefore, it can be assumed that during the journey of a cargo train the source of the noise under the bridge is the deck slab and girder web vibrations. These interactions cause vibrations of the bridge components, which constitute the source of the sound. The coherence between the vibrations of the deck and girder web is definitely smaller during the journey of a passenger train (Figure 13). Passenger trains are lighter and do not induce vibrations in the bridge structure, which may cause additional sound emission.

**Figure 14:**
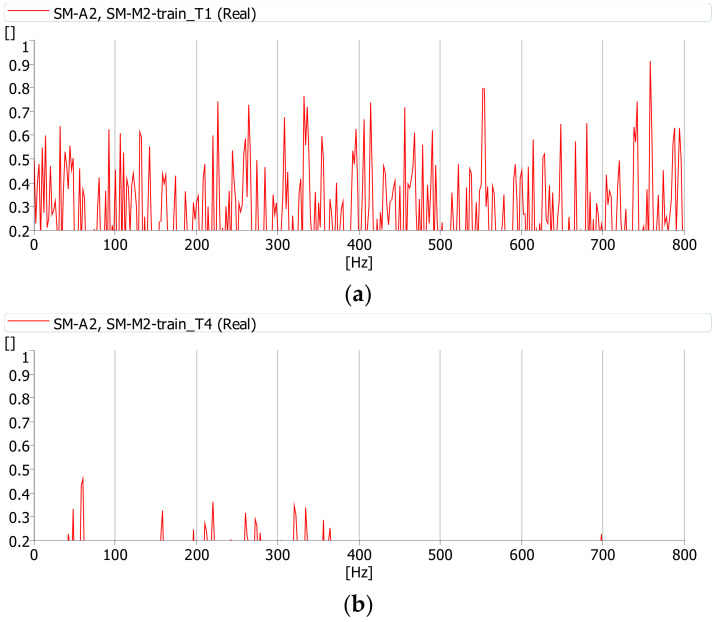
Coherence function between acoustic pressure at point M2 and vibrations at point A2 during the passage of a: (**a**) cargo train (T1), (**b**) passenger train (T4).

Figure 14 shows the coherence function between vibrations of the girder web (m.p. A2) and the acoustic pressure in point M2 for the passage of cargo and passenger trains. It can be seen that the only passage of a cargo train causes vibrations which contribute to the noise.

The measurements and analyses were performed several times at the same measuring points, but with different railway vehicles passing. Coherence functions are similar for different types of trains (cargo, passenger). Therefore, it can be concluded that acoustic phenomena determined during a normal operation of the bridge result primarily from the type of structure and the type of train. Cargo trains have a much greater impact on the environment.

## 6. Conclusions

The conducted vibration analysis allows the following conclusions to be drawn. The tested steel plate girder bridges with concrete deck and tracks laid on the ballast meet the standard requirements for maximum vertical vibration of the structure.

However, this type of bridge may pose a threat to environment. The noise next to the tested structure in most cases did not differ significantly from the noise next to the track, beyond the bridge when a passenger train passes by. During the passage of passenger trains, noise increases max. 2.5 dB. During the passage of cargo trains, noise increases max. 5.8 dB.

In the analysed case, the noise level under the bridge was max. 4.6 dB higher than the noise next to the railway line beyond the bridge, only when a cargo train passes by. The increase is observed in the range of low and medium frequencies, up to 800 Hz. This phenomenon was caused by vibrations of bridge structural elements. The passage of a cargo train excited vibrations in large-surface elements, in this case the deck and girder web. The analysis in narrow frequency bands allowed to confirm the high compatibility of deck and girder web vibrations and sounds. A small agreement or no agreement exists between vibrations of the deck, girder web, and acoustic pressure under the bridge when a passenger train passes by.

From the acoustic point of view, the tested bridge does not pose a threat because it is located outside the built-up area. However, this type of structure located in urbanized areas can be a nuisance to the environment.

## Figures and Tables

**Table 1:** Results of vibration acceleration measurements.

Train Symbol	Train Description	Passage Speed [km/h]	Maximum Vertical Acceleration [m/s^2^]	
			Deck Slab (m.p. A1)	Flange of the Girder (m.p. A3)
T1	Cargo train with M62 locomotive and 24 cars	40	0.50	0.87
T2	Railbus PESA EN62A	115	0.21	0,27
T3	Passenger train with EP-09 locomotive and 7 cars	120	0.20	0.26
T4	Passenger train with EP-07 locomotive and 9 cars	110	0.44	0.71
T5	Passenger train with EP-07 locomotive and 7 cars	125	0.36	0.46
T6	Passenger train with EU-07 locomotive and 7 cars	70	0.15	0.17
T7	Railbus PESA EN62A	120	0.10	0.11
T8	Passenger train with NEWAG locomotive and 6 cars	130	0.24	0.28
T9	Passenger train with EP-07 locomotive and 7 cars	140	0.31	0.36
T10	Cargo train with M62 locomotive and 20 cars	40	0.61	0.85

**Table 2:** Results of noise measurements.

Train Symbol	Noise Level [dB(A)]			Differences [dB]		
	Beyond the Bridge (m.p. M1)	Beside the Bridge (m.p. M2)	Under the Bridge (m.p. M3)	M2-M1	M3-M1	M3-M2
T1	79.1	84.9	83.7	5.8	4.6	−1.2
T2	77.2	79.5	78.2	2.3	1.0	−1.3
T3	94.5	97.0	84.9	2.5	−9.6	−12.1
T4	93.6	96.0	83.4	2.4	−10.2	−12.6
T5	97.1	98.6	86.3	1.5	−10.8	−12.3
T6	89.0	91.0	84.2	2.0	−4.8	−6.8
T7	79.1	80.2	79.7	1.1	0.6	−0.5
T8	95.1	96.3	84.2	1.2	−10.9	−12.1
T9	96.3	98.1	85.2	1.8	−11.1	−12.9
T10	78.3	82.2	82.8	3.9	4.5	0.6

## References

[1] Gulvanessian H. (2001). EN 1990 Eurocode—Basis of Structural Design. Civ. Eng..

[2] Kilikevicius A., Fursenko A., Jurevicius M., Kilikeviciene K., Bureika G. (2017). Analysis of parameters of railway bridge vibration caused by moving rail vehicles. Meas. Control..

[3] Kozuma Y., Nagakura K. (2012). An investigation on vibratory and acoustical characteristics of concrete Bridge for Shinkansen. Noise Vib. Mitig. Rail Trans. Sys..

[4] Cheng Y.S., Au F.T.K., Cheung Y.K. (2001). Vibration of railway bridges under a moving train by using bridge-trackvehicle element. Eng. Struct..

[5] Li Q., Wu D.J. Test and evaluation of high frequency vibration of a U-shaped girder under moving trains. Proceedings of the 9th International Conference on Structural Dynamics.

[6] Costley R.D., Diaz-Alvares H., McKenna M.H., Jordan A.M. (2015). Vibration and Acoustic Analysis of Trussed Railroad Bridge Under Moving Loads. J. Vib. Acoust..

[7] CEN (2005). EN 1993 Eurocode—Design of Steel Structures.

[8] UIC Code 717R (2010). Recommendations for the Design of Bridges to Satisfy Track Requirements and Reduce Noise Emissions.

[9] Thompson D. (2009). Bridge noise. Railway Noise and Vibrations: Mechanisms, Modelling and Means of Control.

[10] Hardy A.E.J. (1999). Noise from railway bridges. Proc. Instn. Mech. Engrs Part F J. Rail Rapid Transit..

[11] Shiva A., Purohit R., Rana R.S., Koli D.K. Noise and Vibration Emissions of Railway Bridges. Proceedings of the 5th International Conference of Materials Processing and Characterization.

[12] Li X., Yang D., Chen G., Li Y., Zhang X. (2016). Review of recent progress in studies on noise emanating from rail transit bridges. J. Mod. Transp..

[13] Janas L. (2018). The Noise Analysis in the Vicinity of Rail Plate Girder Bridges with Different Types of Construction. Rocz. Ochr. Sr..

[14] Janas L. (2019). Railway Plate Girder Bridges as a Source of Noise: Examples Selected. IOP Conf. Ser. Mater. Sci. Eng..

[15] Li Z.G., Wu T.X. (2012). Estimation of Vibration Power Flow to and Sound Radiation from a Railway Concrete Viaduct Due to Vehicle/Track Interaction. Noise Vib. Mitig. Rail Trans. Sys..

[16] Liu Q., Li X., Zhang X., Zhang Z. Structure-borne noise study of composite steel bridge on high–speed railway. Proceedings of the 9th International Conference on Structural Dynamics 2014, EURODYN 2014.

[17] Oostdijk J., Weekenstroo T., Vercammen M. Noise prediction of a steel-concrete railway bridge using a FEM. Proceedings of the EuroNoise 2015.

[18] Janas L. (2019). Identification and analysis of noise sources in a plate girder railway bridge with orthotropic deck. Rocz. Ochr. Sr..

[19] Zvolenský P., Grenčík J., Pultznerová A., Kašiar L. (2017). Research of noise emission sources in railway transport and effective ways of their reduction. MATEC Web Conf..

[20] Saito M., Sugimoto I., Sasaki E. (2015). Experimental study on noise reduction effect of installing concrete deck on existing steel girders. Int. J. Steel Struct..

[21] Song X., Li Q. (2018). Numerical and experimental study on noise reduction of concrete LRT bridges. Sci. Total. Environ..

